# Community-acquired pneumonia in hospitalized adults: long-term morbidities and their risk factors

**DOI:** 10.1186/s12879-025-11186-w

**Published:** 2025-07-01

**Authors:** Jin H. Han, J. Jackson Resser, Adrienne Baughman, Carlos G. Grijalva, Jakea Johnson, Karen F. Miller, Craig S. Roberts, Kelly D. Johnson, Thomas Weiss, Wesley H. Self, for the PNEUMO Study Investigators

**Affiliations:** 1 Department of Emergency Medicine, Vanderbilt University Medical Center, 2215 Garland Ave, Light Hall 203, Nashville, TN 37232-4700 USA; 2 Geriatric Research, Education, and Clinical Center, Tennessee Valley Healthcare System, Nashville, TN USA; 3 Department of Biostatistics, Vanderbilt University Medical Center, Nashville, TN USA; 4 Vanderbilt Institute for Clinical and Translational Research, Vanderbilt University Medical Center, Nashville, TN USA; 5 Department of Health Policy, Vanderbilt University Medical Center, Nashville, TN USA; 6 Value & Implementation, Outcomes Research, Merck & Co., Inc, Rahway, NJ USA

**Keywords:** Pneumonia, Delirium, *Streptococcus pneumoniae*, Morbidity, Activities of daily living, Cognition, Employment, Quality of life

## Abstract

**Background:**

The long-term morbidity associated with community-acquired pneumonia (CAP) and the risk factors associated with this long-term morbidity are unclear. In adults hospitalized for CAP, we sought to quantify the impact of CAP on loss of function, cognitive impairment, loss of employment, and quality of life six months after hospitalization for CAP and identify risk factors for these adverse outcomes.

**Design:**

This prospective cohort study is an analysis of the Pneumococcal Pneumonia Epidemiology, Urine serotyping, and Mental Outcomes (PNEUMO) study population conducted at an academic, quaternary care hospital. Patients aged ≥ 50 years old and hospitalized for CAP between 2018 and 2020 were included in this analysis. Risk factors, including demographics, pre-illness status, comorbidities, delirium at enrollment, and pneumococcal etiology, were considered. Follow-up was conducted by phone six months after hospitalization for CAP to assess basic and instrumental activities of daily living (ADL), cognition, employment, and quality of life. Proportional odds logistic regression was performed to evaluate the association between potential risk factors and long-term morbidity.

**Results:**

Of the 296 patients included in this analysis, 12.8% lost the ability to perform at least 1 basic ADL, 22.8% lost the ability to perform at least 1 instrumental ADL, 41.6% met criteria for cognitive impairment, 58.7% had a loss of employment, and 23.6% had decreased quality of life at 6-months. Poorer pre-illness ability to perform instrumental ADLs and quality of life, lower education, female sex, former and current tobacco use, past history of dementia, and delirium were associated with worsening 6-month outcomes, with the exception of employment.

**Conclusions:**

Adult patients hospitalized for CAP suffered from significant morbidity at 6 months after discharge. Strategies are needed to prevent or mitigate these adverse outcomes, especially among those at higher risk.

**Supplementary Information:**

The online version contains supplementary material available at 10.1186/s12879-025-11186-w.

## Background

In the United States, pneumonia accounted for 1.7 million ED visits [1], 1.2 million hospitalizations [2], and 55,000 deaths annually [3] before the COVID-19 pandemic. Pneumonia is the fourth leading cause of death worldwide [3]. However, long-term morbidity associated with community-acquired pneumonia (CAP) is not fully understood. The COVID-19 pandemic highlighted that acute respiratory infections can cause severe long-term morbidity, such as loss of the ability to perform activities of daily living (ADLs), cognitive impairment, loss of employment, and poor quality of life months to years after the acute illness [4–6].

There is emerging evidence that other acute respiratory illnesses are associated with long-term morbidity. Influenza has been shown to negatively impact quality of life after acute infection [7, 8]. Respiratory syncytial virus (RSV) has also been associated with poorer quality of life [9], physical disability, decreased ability to perform leisure activities, and increased difficulty in working their job [10]. Few studies, however, have evaluated the effect of non-COVID CAP on long-term patient outcomes. Furthermore, to our knowledge, no studies have evaluated risk factors for long-term morbidity for non-COVID-19 pneumonia. Identifying risk factors may help develop strategies to prevent or mitigate neuropsychological dysfunction and disability after non-COVID-19 pneumonia.

To address these gaps in knowledge, we sought to: (1) characterize the burden of the following long-term morbidities 6 months after hospitalization for CAP: loss in the ability to perform basic and instrumental ADLs, cognitive impairment, loss of employment, and decreased quality of life; and (2) identify risk factors associated with these long-term morbidities.

## Methods

### Study design and setting

The Pneumococcal Pneumonia Epidemiology, Urine serotyping, and Mental Outcomes (PNEUMO) study is a multicenter, prospective observational surveillance study designed to understand the epidemiology of CAP [11]. The current analysis focuses on 6-month outcomes, including function, cognition, employment, and quality of life, among the PNEUMO cohort enrolled at Vanderbilt University Medical Center between September 1, 2018 and August 31, 2020. The study was funded by Merck Sharp & Dohme LLC, a subsidiary of Merck & Co., Inc., Rahway, NJ, USA (MSD) and approved by the Institutional Review Board at Vanderbilt University Medical Center. All methods were carried out following the Helsinki Declarations and guidelines and regulations of the Vanderbilt University Medical Center Institutional Review Board.

### Selection of participants

Details of the PNEUMO study’s eligibility criteria have been previously reported [11]. In brief, patients were enrolled if they were: 18 years old or older; admitted to the hospital; had clinical signs and/or symptoms consistent with an acute respiratory infection; had radiographic evidence of pneumonia; and were able to provide a urine sample. Patients who developed pneumonia > 48 h after hospital admission were considered to have hospital-acquired pneumonia and were not enrolled. For this analysis of long-term outcomes, patients were included if they: were 50 years old or older; enrolled at Vanderbilt University Medical Center (one of the participating sites of the overall multicenter program); did not test positive for SARS-CoV2 during their acute illness; and did not opt out of follow-up assessments after hospital discharge. Only patients who were 50 years or older were considered for enrollment in the long-term outcomes analysis to maintain feasibility; because the majority of these patients were not critically ill, patients who are < 50 years are less likely to suffer from long-term morbidity after CAP.

### Risk factors for long-term morbidity

Patient and disease characteristics present at the time of hospitalization for CAP were assessed as potential risk factors for long-term morbidity following the acute phase of CAP. The following 12 potential risk factors were included: 1) age, 2) sex, 3) past history of dementia, 4) pre-illness Barthel Index, 5) pre-illness Lawton Instrumental ADL scale, 6) pre-illness EQ-5D-5L, 7) years of education, 8) Charlson comorbidity index, 9) CURB-65 (pneumonia severity of illness), 10) tobacco smoking status, 11) pneumococcal etiology, and 12) delirium at enrollment. The Barthel Index characterizes 10 basic ADLs (i.e., feeding, bathing, toileting, etc.) and ranges from 0 (complete dependence) to 20 (complete independence) [12]. The Lawton Instrumental ADL scale characterizes 8 instrumental ADLs (i.e., using the telephone, shopping, food preparation, etc.) and ranges from 0 (complete dependence) to 8 (complete independence) [13]. The EQ-5D-5L characterizes quality of life and is a 5-question questionnaire that asks the patient to rate their own impairments in mobility, self-care, usual activities, pain/discomfort, and anxiety/depression [14]. EQ-5D-5L index values, validated for the US population, were calculated, and higher scores represented better quality of life [15]. To establish pre-illness baseline Barthel, Lawton, and EQ-5D-5L scores, patients or their informants were asked to retrospectively characterize their ability to perform basic and instrumental ADLs, and quality of life two weeks before the CAP illness. Past history of dementia, Charlson Comorbidity burden [16], and CURB-65 [17] were collected from the electronic health record.

At enrollment, patients were asked by research staff if they currently smoked, formerly smoked, or never smoked. Patients were considered to have a pneumococcal CAP if they had a positive biospecimen culture for *Streptococcus pneumoniae*, a positive BinaxNOW pneumococcal urinary antigen test [18], or a positive pneumococcal serotype-specific urinary antigen detection (SSUAD) assay; methods for pneumococcal testing in this study have been previously described [11]. Delirium was determined at enrollment using the modified Brief Confusion Assessment Method (bCAM) by trained research assistants. The modified bCAM is 82–86% sensitive and 93–96% specific for the identification of delirium compared with a psychiatrist’s diagnosis in older patients and has a kappa of 0.87, indicating very good inter-observer reliability [19].

### Six month outcomes

Patients were called via telephone 6 months (± 2 months) after hospital admission for CAP to assess five outcomes: 1) basic ADLs; 2) instrumental ADLs; 3) global cognition; 4) loss of employment; and 5) quality of life. The threshold to define severe morbidity for each of these outcomes is described below. A decrease in Barthel’s index >  = 2 points compared to pre-illness baseline indicates a loss of one basic ADL. A decrease in Lawton’s Instrumental ADL scale >  = 1 point compared to pre-illness baseline indicates a loss of one instrumental ADL. Global cognition was characterized using the Telephone Montreal Cognitive Assessment (T-MoCA), which ranges from 0 (severe cognitive impairment) to 22 (no cognitive impairment), with a score < 18 indicating the presence of cognitive impairment. Employment was collected using the Outcomes After Critical Illness and Surgery (OACIS) Return to Work survey [20]. Loss of employment was defined as a patient who was either: (1) fully employed prior to the CAP illness and became partially employed or unemployed; or (2) partially employed prior to the CAP illness and became unemployed. A decrease in EQ-5D-5L index value of at least 0.078 compared to pre-illness baseline was considered a clinically important decrease in quality of life [15].

### Data analysis

Measures of central tendency and dispersion were reported as medians and interquartile ranges (interquartile range [IQR]). Frequency and proportions were reported for categorical variables. The proportions of the study population who had a loss of at least 1 basic ADL, a loss of at least 1 instrumental ADL, cognitive impairment, loss of employment, or a decrease in quality of life were reported. For loss of employment, the analysis was performed in a subset of patients who were partially or fully employed prior to the acute CAP illness. The proportion of 6-month cognitive impairment was also reported for a subset of patients who were < 60 years old and < 60 years old without a past history of dementia; these patients are highly likely to be cognitively intact at baseline, and the presence of cognitive impairment in 6 months would likely be new. As an exploratory analysis, the study population was also stratified by pneumococcal testing results into those with pneumococcal etiology and those without pneumococcal etiology. The proportion of patients who experienced morbidity for each of the 5 outcomes using the thresholds defined above was compared between the pneumococcal group and non-pneumococcal group using the Chi-squared test.

To evaluate for risk factors for each of the 5 outcomes, we fitted multivariable proportional odds regression models using the 12 potential risk factors (except where otherwise stated) as independent variables and each outcome as the dependent variable in separate models. Because of the dichotomous nature of loss of employment and the smaller number of events, Firth’s logistic regression was performed to minimize overfitting. Pre-illness dementia and delirium were not included in the loss of employment model because there were only one (0.9%) and four (3.8%) patients with these covariates, respectively. Collinearity between risk factors was assessed using variance inflation factors (VIF). A VIF less than 10 indicated no collinearity [21]. To maintain consistent directionality where an aOR < 1.00 indicates a worse outcome, the aORs of pre-illness Barthel, Lawton, EQ-5D-5L, and year of education were inverted. Adjusted odds ratios (aOR) with their 95% confidence intervals (95%CI) were reported. A p-value of < 0.05 was considered statistically significant. All statistical analyses were performed using R (R Foundation for Statistical Computing, R version 4.4.0, Vienna, Austria).

## Results

During the 2-year enrollment period for this analysis, 1286 patients were enrolled in the PNEUMO study at the Vanderbilt site; of these, 374 were less than 50 years old, 346 opted out of follow-up, 92 died (82 during hospitalization and 10 post-hospital discharge) before the 6-month assessment, and 178 did not complete the 6-month follow-up, leaving 296 patients available for this analysis. Compared to those included in the analysis, patients who were eligible for but did not complete the 6-month follow-up were similar in terms of age, ethnicity, race, educational attainment, comorbidity burden, pre-illness dementia, pre-illness functional impairment, pre-illness quality of life, severity of illness, and pneumococcal etiology, and delirium at enrollment (eTable). However, those who declined 6-month follow-up were more likely to be smokers.

Patient characteristics of those included in this analysis are listed in Table 1. The median (IQR) age was 65.6 (59.6, 73.0) years old; 122 (41.2%) were female, 41 (13.9%) were Black, and 38 (12.8%) had pneumococcal disease.

**Table 1:** Patient characteristics

Variable	Enrolled, *n* = 296
Median (IQR) age, years	65.6 (59.6, 73.0)
Female sex, n (%)	122 (41.2%)
Ethnicity	
Hispanic/LatinX, n (%)	270 (92.2%)
Non-Hispanic/LatinX, n (%)	2 (0.7%)
Declined to Answer/Unknown, n (%)	21 (7.2%)
Race	
White, n (%)	247 (83.4%)
Black, n (%)	41 (13.9%)
Asian, n (%)	0 (0.0%)
American Indian or Alaskan Native, n (%)	0 (0.0%)
Native Hawaiian or Other Pacific Islander, n (%)	0 (0.0%)
Multiple Race, n (%)	1 (0.3%)
Tobacco use	
Never smoked, n (%)	145 (49.0%)
Previous smoker, n (%)	128 (43.2%)
Current smoker, n (%)	23 (7.8%)
Education	
Did not complete high school, n (%)	33 (11.1%)
High school graduate, no college, n (%)	87 (29.4%)
Some college, no degree, n (%)	66 (22.3%)
Associate degree, n (%)	24 (8.1%)
Bachelors degree, n (%)	48 (16.2%)
Advanced degree, n (%)	37 (12.5%)
Missing, n (%)	1 (0.3%)
Median (IQR) Charlson Comorbidity Index	5 (3, 7)
Pre-illness dementia, n (%)	15 (5.1%)
Median (IQR) pre-illness Barthel	19 (17, 20)
Median (IQR) pre-illness Lawton	7 (5, 8)
Median (IQR) pre-illness EQ-5D-5L	0.83 (0.58, 1.00)
Median (IQR) CURB-65	1 (1, 2)
Median (IQR) hospital length of stay, days	3 (2, 6)
ICU admission, n (%)	10 (3.9%)
Pneumococcal disease, n (%)	38 (12.8%)
Delirium at enrollment, n (%)	23 (8.5%)

IQR, interquartile range

The burden of 6-month morbidity after CAP hospitalization is shown in Table 2. Compared to pre-illness status, 12.8% of patients lost the ability to perform at least 1 basic ADL, 22.8% lost the ability to perform at least 1 instrumental ADL, 58.7% had a loss of employment, and 23.6% had poorer quality of life. Additionally, 41.6% met criteria for cognitive impairment. Cognitive impairment was identified in 40.1% of those without a past history of dementia, and 37.5% of those who were less than 60 years old.

**Table 2:** Basic activities of daily living, instrumental activities of daily living, cognition, employment, and quality of life at 6 months

Outcomes at 6-months	Total n	Median (IQR) or n (%)
Median (IQR) Barthel Index	296	20.00 (18.00, 20.00)
Change from baseline Barthel Index > = 2, n (%)	296	38 (12.8%)
Median (IQR) Lawton Instrumental ADL	295	8.00 (5.50, 8.00)
Change from baseline Lawton Instrumental ADL > = 1, n (%)	294	67 (22.8%)
Median (IQR) T-MoCA	255	18 (16, 20)
T-MoCA < 18, n (%)	255	106 (41.6%)
Incident cognitive impairment among patients without a past history of dementia, n (%)	247	99 (40.1%)
Median (IQR) T-MoCA among patients 60 years old	72	18 (16, 20)
T-MoCA < 18 among patients < 60 years old, n (%)	72	27 (37.5%)
Incident cognitive impairment among patients without a past history of dementia among patients < 60 years old), n (%)	72	27 (37.5%)
Loss of employment among those who were employed before the illness		
Any loss of employment, n (%)	109	64 (58.7%)
Full to partial employment, n (%)	89	6 (6.7%)
Full to unemployed, n (%), n (%)	89	47 (52.8%)
Partial to unemployed, n (%)	20	11 (55.0%)
Median (IQR) EQ-5D-5L Index Score	296	0.75 (0.50, 0.93)
Change from baseline EQ-5D-5L > 0.078, n (%)	296	70 (23.6%)

IQR, interquartile range; ADL, activities of daily living, T-MoCA, Telephone Montreal Cognitive Assessment

The burden of 6-month morbidity stratified by pneumococcal vs non-pneumococcal status is shown in Table 3. No significant differences in 6-month outcomes were observed between patients in the pneumococcal and non-pneumococcal etiology groups.

**Table 3:** Basic ADLs, instrumental ADLs, cognition, employment, and quality of life at 6 months, stratified by pneumococcal etiology

Outcomes at 6-months	Pneumococcal, *n* = 38	Pneumococcal Median (IQR) or n (%)	Non-Pneumococcal, *n* = 258	Non-Pneumococcal Median (IQR) or n (%)	*p*-value
Median (IQR) Barthel Index	38	20 (18, 20)	258	20 (18, 20)	
Severe morbidity: Change from baseline Barthel Index > = 2, n (%)	38	7 (18.4%)	258	31(12.0%)	0.27
Median (IQR) Lawton Instrumental ADL	37	7 (6, 8)	258	8 (5.25, 8)	
Severe morbidity: Change from baseline Lawton Instrumental ADL > = 1, n (%)	37	12 (32.4%)	257	55 (21.4%)	0.13
Median (IQR) T-MoCA	28	18 (14.75, 20)	227	19 (16, 20)	
T-MoCA < 18	28	13 (46.4%)	227	93 (41.0%)	0.62
Severe morbidity: Incident cognitive impairment among patients without a past history of dementia, n (%)	27	12 (44.4%)	220	87 (39.5%)	
Median (IQR) T-MoCA among patients 60 years old	14	19.5 (17, 20.75)	58	18 (16, 20)	
T-MoCA < 18 among patients < 60 years old	14	5 (35.7%)	58	22 (37.9%)	
Incident cognitive impairment among patients without a past history of dementia among patients < 60 years old), n (%)	14	5 (35.7%)	58	22 (37.9%)	
Loss of employment among those who were employed before the illness [severe morbidity: any loss of employment], n (%)	20	13 (65.0%)	89	51 (57.3%)	0.53
Sub-categories of loss of employment					
Full to partial employment	17	0 (0.0%)	72	6 (8.3%)	
Full to unemployed	17	11 (64.7%)	72	36 (50.0%)	
Partial to unemployed	3	2 (66.7%)	17	9 (52.9%)	
Median (IQR) EQ-5D-5L Index Score	38	0.72 (0.56, 0.89)	258	0.75 (0.49, 0.93)	
Severe morbidity: Change from baseline EQ-5D-5L > 0.078, n (%)	38	7 (18.4%)	258	63 (24.4%)	0.42

Reported *p*-values were calculated using the Chi-square test to show the difference in proportions in the pneumococcal group vs non-pneumococcal group for patients who met criteria for severe morbidity for each of the 5 outcomes. IQR, interquartile range; ADL, activities of daily living, T-MoCA, Telephone Montreal Cognitive Assessment

**Fig. 1:**
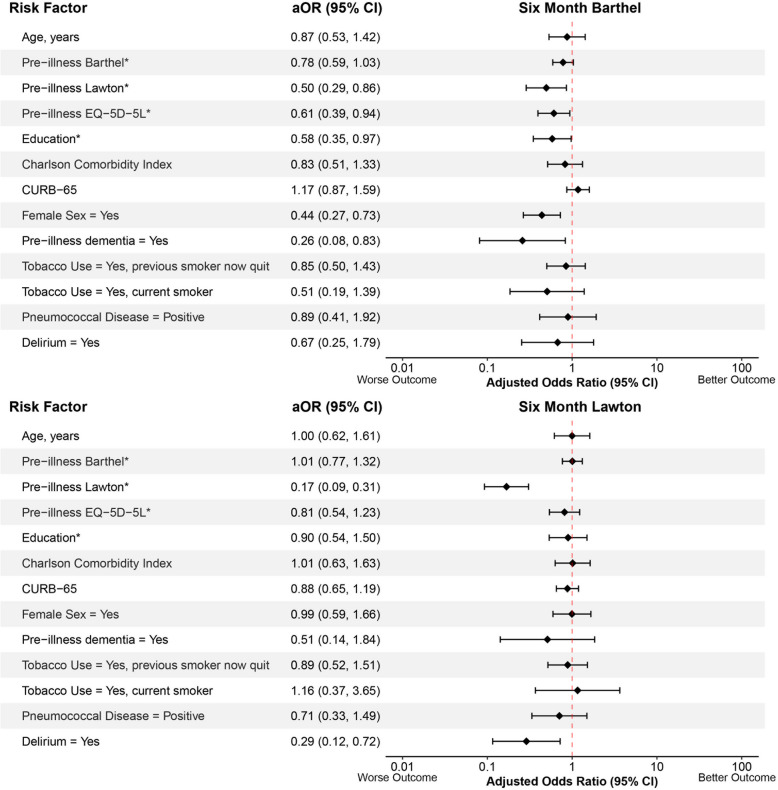
Forest plots showing the association between potential risk factors and 6-month Barthel (basic activities of daily living, continuous) and Lawton (instrumental activities of daily living, continuous) scores using proportional odds logistics regression. Adjusted odds ratios (aOR) with their 95% confidence intervals (95% CI) are presented. *The aORs of pre-illness Barthel, Lawton, EQ-5D-5L, and year of education are inverted to keep directionality consistent where an aOR < 1.00 indicates a worse outcome

**Fig. 2:**
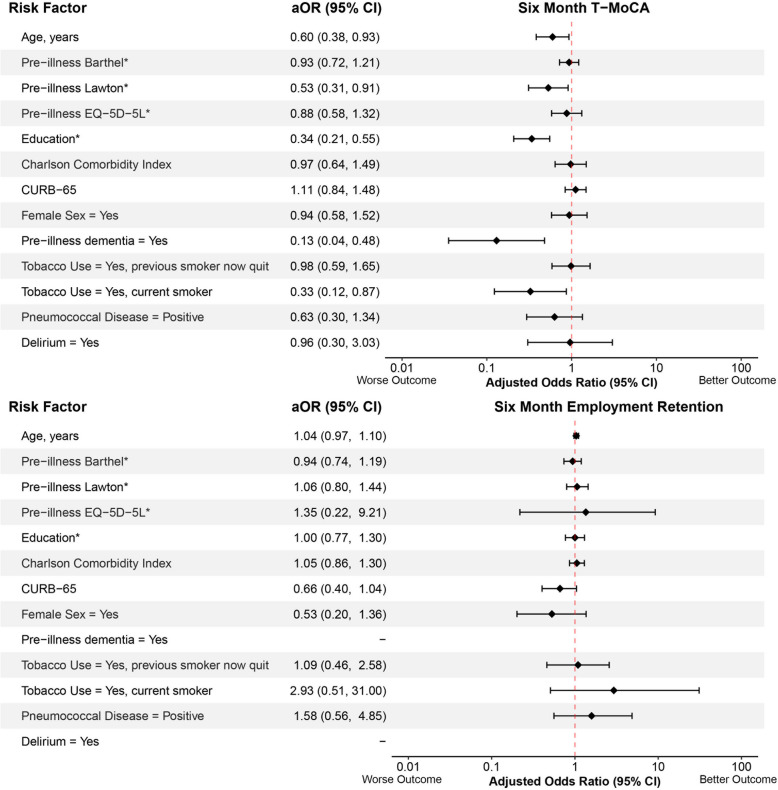
Forest plots showing the association between potential risk factors and 6-month Telephone Montreal Cognitive Assessment (T-MoCA, global cognition, continuous) scores and employment retention (dichotomous). For the six-month employment retention model, a subset of patients who were employed prior to the community-acquired pneumonia illness were included in this analysis. The pre-illness dementia and delirium variables were not incorporated into the model because few patients had these risk factors. Adjusted odds ratios (aOR) with their 95% confidence intervals (95% CI) are presented. *The aORs of pre-illness Barthel, Lawton, EQ-5D-5L, and year of education are inverted to keep directionality consistent where an aOR < 1.00 indicates a worse outcome

**Fig. 3:**
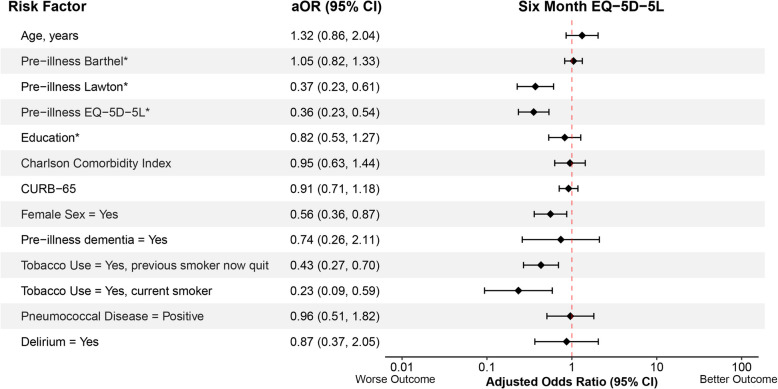
Forest plots showing the association between potential risk factors and 6-month EQ-5D-5L (quality of life, continuous). Adjusted odds ratios (aOR) with their 95% confidence intervals (95% CI) are presented. *The aORs of pre-illness Barthel, Lawton, EQ-5D-5L, and year of education are inverted to keep directionality consistent where an aOR < 1.00 indicates a worse outcome

The associations between potential risk factors and outcomes 6 months after CAP hospitalization are shown in Figs. 1, 2 and 3. All VIFs were less than 3, indicating no evidence of substantial collinearity between risk factors. Lower pre-illness Lawton instrumental ADLs (aOR = 0.50, 95% CI: 0.29, 0.86), lower pre-illness EQ-5D-5L index values (aOR = 0.61, 95% CI: 0.39, 0.94), fewer years of education (aOR = 0.58, 95% CI: 0.35, 0.97), female sex (aOR = 0.44, 95% CI: 0.26, 0.73), and pre-illness dementia (aOR = 0.26, 95% CI:0.08, 0.83) were significantly associated with lower 6-month Barthel’s Index (poorer ability to perform basic ADLs). Lower pre-illness Lawton instrumental ADLs (aOR = 0.17, 95% CI 0.09, 0.31) and delirium (aOR = 0.29, 95% CI: 0.12, 0.72) were significantly associated with lower 6-month Lawton’s instrumental ADL scores (poorer ability to perform instrumental ADLs). Older age (aOR = 0.60, 95% CI: 0.38, 0.93), lower pre-illness Lawton instrumental ADLs (aOR = 0.53, 95%CI: 0.31, 0.91), fewer years of education (aOR = 0.34, 95% CI: 0.24, 0.55), pre-illness dementia (aOR = 0.13, 95% CI: 0.04, 0.48), and current tobacco use (aOR = 0.33, 95% CI: 0.12, 0.87) were significantly associated with lower 6-month T-MoCA scores (poorer global cognition). Lower pre-illness Lawton instrumental ADLs (aOR = 0.37, 95% CI: 0.23, 0.61), lower pre-illness EQ-5D-5L index values (aOR = 0.36, 95% CI: 0.23, 0.54), female sex (aOR = 0.56, 95% CI: 0.36, 0.87), previous tobacco use (aOR = 0.43, 95% CI: 0.27, 0.70), and current tobacco use (aOR = 0.23, 95% CI: 0.09, 0.59) were significantly associated with lower 6-month EQ-5D-5L index values (poorer quality of life). No risk factors were significantly associated with loss of employment.

## Discussion

A considerable proportion of patients who were 50 years or older and were hospitalized for CAP lost their ability to perform basic and instrumental ADLs, had a loss of employment, and had poorer quality of life compared to their pre-illness baseline. In addition, 40% met criteria for cognitive impairment at 6 months, and this proportion was consistently high in those without pre-illness dementia status and who were 50–65 years old. Identifying risk factors for these devastating outcomes is a critical first step to mitigating these adverse patient outcomes. We observed that lower pre-illness ability to perform instrumental ADLs, poorer pre-illness quality of life, lower educational attainment, female sex, pre-illness dementia, former and current tobacco use, and delirium were significantly associated with long-term morbidity after CAP hospitalization.

Developing interventions and strategies to prevent the sequelae associated with CAP in these high-risk patients during and after hospitalization are needed. Based on observational cohort studies, these interventions may include intensive physical, occupational, and speech therapy during and after hospitalization, but randomized trials are needed to confirm the benefits of these therapies [22–24]. We also observed that delirium was associated with worsening ability to perform 6-month instrumental ADLs. Having an intact ability to perform instrumental ADLs is vital to living normal daily life. Thus, patients hospitalized for pneumonia may benefit from non-pharmacologic multi-component delirium prevention protocols [25, 26]. These interventions include enhancing mobilization and avoiding tethers, normalizing sleep–wake cycles that are often reversed in delirium, using glasses and hearing aids to correct sensory impairment, and performing cognitive stimulating activities.

Our findings are consistent with previous studies, which reported that patients who were hospitalized for CAP had difficulty performing their ADLs [27–29], suffered from cognitive impairment [30–32], and had poor quality of life [33] months to years after the acute illness. The effect of CAP on long-term employment, however, is not well established. We observed that among those who were employed prior to the CAP hospitalization, 58% experienced a loss of employment at 6 months. Strikingly, of those who were fully employed prior to the CAP hospitalization, 52% were not employed at 6 months. These findings extend the work of Daniel et al., who reported that 34% of 108 patients who were hospitalized for CAP did not return to work at 4 weeks [34]. Furthermore, in 250,000 employees, Kleinman et al. observed that CAP nearly doubled employees' turnover rate compared to non-pneumonia controls [33].

Of note, the subgroup of patients with CAP caused by pneumococcal infection similarly experienced substantial 6-month morbidity as those without pneumococcal infection. There was also a trend towards higher losses of instrumental ADLs in the pneumococcal CAP group compared to non-pneumococcal CAP (32.4% vs 21.4%), but this association did not reach statistical significance (*p*-value = 0.13). Pneumococcal disease is likely the most common bacterial etiology of CAP in the world [35] and comprised 12.8% of our cohort. Pneumococcal vaccination may be an important strategy to reduce long-term morbidity by preventing illness secondary to pneumococcal disease or attenuating the severity of illness if the patient develops pneumococcal CAP. To our knowledge, no studies have rigorously studied the effect of pneumococcal vaccines on long-term outcomes after CAP. Reductions in long-term morbidity have been observed with COVID-19 vaccinations after an acute COVID-19 infection [36]. Future studies are needed to evaluate if pneumococcal vaccines will have a similar impact on long-term outcomes.

The strengths of this study include prospective enrollment using eligibility confirmed in real-time at the bedside, assessments of pre-illness ADLs and employment to put 6-month results into context, and systematic use of well-validated tools for assessing 6-month outcomes.

Our study also had limitations. First, our cohort only included hospitalized patients who were ≥ 50 years old, and our findings cannot be generalized to outpatients with milder pneumonia or younger patients. This study was conducted at an academic, quaternary care hospital. Our findings may not be generalizable to community settings. Second, it is unclear how much of the long-term morbidity observed is secondary to the CAP illness versus the hospitalization itself. Future studies should compare the long-term morbidity of patients hospitalized for CAP versus those hospitalized for non-CAP acute respiratory infection or without an acute respiratory illness. Third, it is possible that hospitalized patients with COVID-19 could have been enrolled in our cohort. The first patient with COVID-19 in Tennessee was on March 5, 2020, and Vanderbilt University Medical Center did not routinely start testing for COVID-19 until March 9, 2020. However, we expect this number to be extremely small as the gap in testing occurred early in the pandemic when the incidence of COVID-19 in TN was 17 per 100,000 [37]. Fourth, although our risk factor analyses applied multivariable regression models that accounted for measured factors, it may still be affected by residual confounding. For example, we did not account for pre-illness physical function and social support, which may confound the association between the risk factors and 6-month outcome but were not directly measured. In addition, to account for baseline cognition, we used a past history of dementia, which may not have sufficiently accounted for pre-illness cognition. Fifth, patients with cognitive impairment may have inaccurately characterized their abilities to perform ADLs. However, patients with cognitive impairment tend to overestimate their functional abilities, biasing our results toward the null [38]. Sixth, a significant proportion of eligible patients did not complete the 6-month assessments (e.g., refused to participate or died), potentially introducing selection bias. However, patients who did not complete 6-month follow-up were similar in age, sex, race, comorbidity burden, and severity of illness compared to those in the analysis. Lastly, our sample size of 296 patients with 6-month outcomes was not large enough to definitively establish the association between some baseline characteristics, including pneumococcal disease, and 6-month outcomes.

In conclusion, a considerable proportion of adults hospitalized for CAP lost their ability to perform basic and instrumental ADLs, had poor quality of life, and had a loss of employment at 6 months compared to their pre-illness status. A substantial proportion met the criteria for cognitive impairment at 6 months, even those without a past history of dementia and who were younger than 60 years old. Delirium is a potentially modifiable risk factor associated with long-term morbidity. Additional research evaluating strategies to prevent the sequelae of CAP should be developed.

## Supplementary Information

Supplementary Material 1.

## Data Availability

The data will not be shared.

## Abbreviations

95%CI: 95% Confidence intervals
ADL: Activities of daily living
aOR: Adjusted odds ratio
bCAM: Brief Confusion Assessment Method
CAP: Community- acquired pneumonia
IQR: Interquartile range
MSD: Merck Sharp & Dohme LLC
PNEUMO: Pneumococcal Pneumonia Epidemiology, Urine serotyping, and Mental Outcomes
SSUAD: Serotype-specific urinary antigen detection
T-MoCA: Telephone Montreal Cognitive Assessment
