# Supplementary material for: Community-acquired pneumonia in hospitalized adults: long-term morbidities and their risk factors
Source: BMC Infect Dis. 2025 Jul 1;25:826. doi: 10.1186/s12879-025-11186-w (PMC12211784; doi:10.1186/s12879-025-11186-w)
Supplement: Supplementary file 1 — Supplementary Material 1. [file 12879_2025_11186_MOESM1_ESM.docx]

**eTable.** Patient characteristics of those included in this long-term outcome analysis and those who enrolled in the in-hospital study but declined the follow-up assessment at 6 months. IQR, interquartile range.

| **Variable** | **Included in long-term outcome assessments, n=296** | **Declined long-term outcome assessments**  **N=178** |
| --- | --- | --- |
| Median (IQR) age, years | 65.6 (59.5, 73.0) | 63.7 (57.6, 71.3) |
| Female Sex, n (%) | 122 (41.2%) | 83 (46.6%) |
| Ethnicity |  |  |
| Hispanic/LatinX, n (%) | 270 (92.2%) | 1 (0.6%) |
| Non-Hispanic/LatinX, n (%) | 2 (0.7%) | 171 (97.2%) |
| Declined to Answer/Unknown, n (%) | 21 (7.2%) | 4 (2.3%) |
| Race |  |  |
| White, n (%) | 247 (83.4%) | 141 (79.2%) |
| Black, n (%), n (%) | 41 (13.9%) | 32 (18.0%) |
| Asian, n (%) | 0 (0.0%) | 0 (0.0%) |
| American Indian or Alaskan Native, n (%) | 0 (0.0%) | 0 (0.0%) |
| Native Hawaiian or Other Pacific Islander, n (%) | 0 (0.0%) | 1 (0.6%) |
| Multiple Race, n (%) | 1 (0.3%) | 3 (1.7%) |
| Tobacco use |  |  |
| Never smoked, n (%) | 145(49.0%) | 64 (36.0%) |
| Previous smoker, n (%) | 128 (43.2%) | 73 (41.0%) |
| Current smoker, n (%) | 23 (7.8%) | 541(23.0%) |
| Median (IQR) Charlson Comorbidity Index | 5 (3, 7) | 5.00 (3.00, 7.00) |
| Education |  |  |
| Did not complete high school, n (%) | 33 (11.2%) | 22 (12.8% |
| High school graduate, no college, n (%) | 87 (29.5%) | 53 (30.8% |
| Some college, no degree, n (%) | 66 (22.4%) | 39 (22.7%) |
| Associate degree, n (%) | 24 (8.1%) | 15 (8.7%) |
| Bachelors degree, n (%) | 48 (16.3%) | 19 (11.0%) |
| Advanced degree, n (%) | 37 (12.3%) | 24 (14.0%) |
| Missing, n (%) | 1 (0.3%) | 6 (3.3%) |
| Median (IQR) Charlson Comorbidity Index | 5 (3, 7) | 5 (3, 6) |
| Pre-illness dementia, n (%) | 15 (5.1%) | 8 (4.5%) |
| Median (IQR) pre-illness Barthel | 18 (17, 20) | 19 (17, 20) |
| Median (IQR) pre-illness Lawton | 7 (5, 8) | 7 (5, 8) |
| Median (IQR) pre-illness EQ-5D-5L | 0.83 (0.58, 1.00) | 0.78 (0.56, 0.91) |
| Median (IQR) CURB-65 | 1 (1, 2) | 1 (1, 2) |
| Median (IQR) hospital length of stay, days | 3 (2, 6) | 4 (2,7) |
| ICU admission, n (%) | 10 (3.9%) | 3 (2.1%) |
| Pneumococcal disease, n (%) | 36 (12.3%) | 23 (12.9%) |
| Delirium at enrollment, n (%) | 22 (8.2%) | 12 (8.5%) |
